# Exploring potential drug targets for SLE through Mendelian randomization and network pharmacology

**DOI:** 10.1371/journal.pone.0316481

**Published:** 2025-01-17

**Authors:** Yanan Xu, Zelin Wang, Tiewen Jia, Shufen Liang

**Affiliations:** Department of Laboratory, The Second Hospital of Shanxi Medical University, Taiyuan, Shanxi, P.R. China; Prime Hospital LLC, UNITED ARAB EMIRATES

## Abstract

**Background:**

Systemic lupus erythematosus (SLE) is a complex and incurable autoimmune disease, so several drug remission for SLE symptoms have been developed and used at present. However, treatment varies by patient and disease activity, and existing medications for SLE were far from satisfactory. Novel drug targets to be found for SLE therapy are still needed.

**Methods:**

Mendelian randomization (MR), an observational study way, was performed to explore potential drug targets for SLE using protein quantitative trait loci (pQTL) from recently published genome-wide association studies (GWAS) of cerebrospinal fluid (CSF) and plasma proteins, which obtained genetic instruments for 154 CSF proteins of 971 participants, and 734 plasma proteins of 23591 participants. Bidirectional Mendelian randomization analysis, colocalization analysis, and phenotype scanning were performed to find key proteins for SLE. In addition, external data verification was implemented to further consolidate the Mendelian randomization findings. Candidate proteins as targets to find drugs and discuss the druggability. Finally, Network pharmacology and molecular docking methods were used to verify the effects of Voclosporin and Cyclosporine on SLE targets. Protein-protein interaction (PPI) and core target analysis of candidate drugs and SLE overlapping targets were performed to identify potential hub targets and interactions. The affinity between drug targets and SLE targets was confirmed by molecular docking.

**Results:**

In the preliminary analysis, we identified four key proteins as possible drug targets in CSF and plasma proteins, included ICAM-1(P = 4.62E-05, OR = 0.90(0.86, 0.95)), sICAM-1(P = 4.62E-05, OR = 0.49(0.35, 0.69)), FCG2B (P = 7.63E-11, OR = 0.57(0.48, 0.67)), PPP3CA; PPP3R1 (P = 5.47E-07, OR = 0.66(0.57, 0.78)). Among them, ICAM1 was detected in both CSF and plasma proteins. By excluding reverse causality, confounding factors, and linkage disequilibrium (LD), we identified PPP3CA; PPP3R1 as novel drug targets for SLE, including Voclosporin and Cyclosporine. Finally, the Drugbank database shows that novel drugs contain 33 targets for treating SLE. PPI suggested that SIRT1, ACE, PTGS2, and BACE1 were pivotal targets for SLE treatment. In addition, the molecular docking showed that the bioactive molecules of Voclosporin and Cyclosporine had a good affinity with the target of SLE.

**Conclusions:**

Our integrative analysis suggested that levels of circulating PPP3CA; PPP3R1 had causal effects on SLE risk and served as potential treatment targets. Moreover, this study provides new evidence for Voclosporin as an SLE treatment through Mendelian randomization and Network pharmacology, and warrants further clinical investigation.

## 1. Introduction

Systemic lupus erythematosus (SLE) is a chronic immune system disorder with abnormalities of immune cells, manifesting as the dysregulated immune system, characterized by overactivated B and T cells attacking normal cells and tissues, which affects multiple organs and systems [1]. According to published epidemiological surveys, the incidence and prevalence of SLE are high in North America (241 per 100,000 people) [2]. The prevalence of SLE is estimated to increase annually. In addition, as a systemic disorder, SLE frequently affects the blood, kidneys, respiratory, nervous, and gastrointestinal systems [3], and the specific etiology and the pathophysiology of SLE have remained elusive. Notably, the clinical presentation of SLE is varied and unpredictable leading to delayed diagnosis and difficult treatment. Previous studies have shown that early detection and medication regimens have substantially improved SLE survival outcomes [4]. Clinical medications generally treat SLE including glucocorticoids, antimalarial agents, nonsteroidal anti-inflammatory drugs (NSAIDs), immunosuppressive agents, and B cell-targeting biologics [1]. Nonetheless, treatment varies by patient and disease activity, and novel drug targets for SLE therapy are still needed.

Proteins are engaged in complex biological regulation in living organisms and generally serve as drug targets for diseases. Because of the blood-brain barrier, circulating proteins are divided into plasma proteins and cerebrospinal fluid (CSF) proteins. On the one hand, plasma proteins play crucial roles in a range of biological processes and represent major druggable targets [5]. On the other hand, it is generally acknowledged that CSF is produced in the brain, and CSF proteins are considered ideal biomarkers for observing disease states in the brain [6]. So plasma proteins and CSF proteins are separately adopted in this study to improve the result accuracy of the drug targets of SLE. pQTL can be divided into two categories: cis-pQTL and trans-pQTL. In contrast to cis-pQTL, which are located near the target protein, trans-pQTL are typically found on another chromosome. These trans-pQTL do not directly affect the gene or gene domain of the target protein. Previous studies have shown that selection of cis-pQTL for IV can reduce the effects of false positives and pleiotropy [7]. Hence, cis-pQTL are considered to have more direct biological effects on proteins.

Mendelian randomization (MR) is a statistical method that utilizes genetic variation as an instrumental variable (IV) to draw the causal relationship between exposure and outcome [8]. Compared with traditional observational studies, MR analysis can reduce both confounding variables and reverse causation because alleles are random allocation during meiosis, and provide better evidence of causal inference [9]. It’s worth noting that IV fulfilled three assumptions: a, IV was strongly associated with exposure and independent of each other (P<5×10^−8^, LD <0.001); b, IV was not associated with the outcome; c, IV was not associated with confounders [10] (S1 Fig). MR analysis has been extensively applied in recent years for the development and repurposing of drug targets [11]. In fact, many of experimental clinical drugs have failed to achieve efficacy in clinical trials. However, if a protein drug target linked with the disease is confirmed by genetic association, its market approval will go up [12].

**Fig 1:**
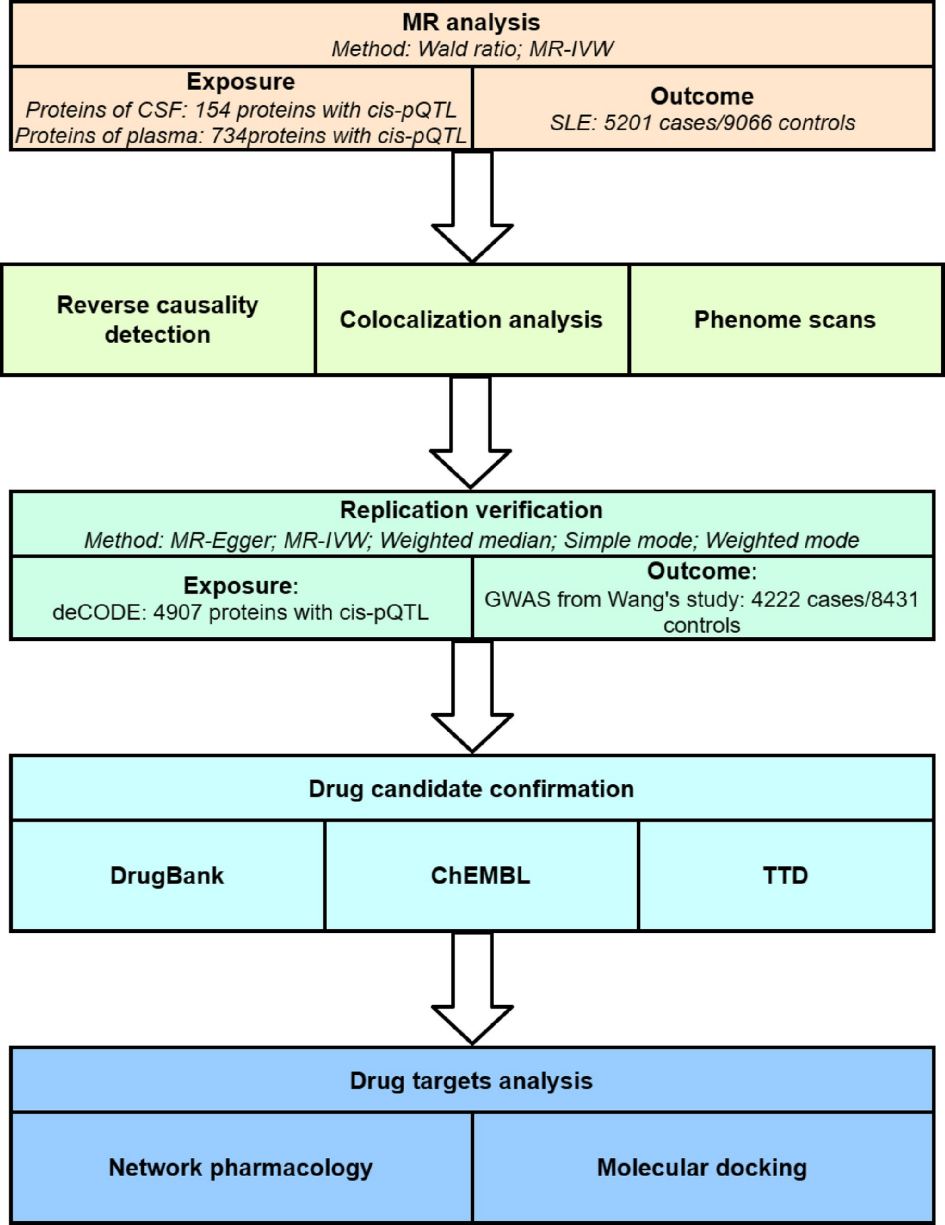
Study design flowchart.

Although there are more and more studies on the treatments of SLE, exploring potential drug targets for SLE through Mendelian randomization in plasma proteins and CSF proteins is still absent. Therefore, the primary objective of this study is to identify potential therapeutic targets for SLE. First, we identify potential causal plasma and CSF proteins for SLE using MR analysis based on GWAS summary data from plasma and CSF proteins. Second, bidirectional MR analysis, colocalization analysis, and phenotype scanning were performed to further validate primary findings. Third, to further ensure the reliability of potential causal proteins, we conducted a replication verification using external data. Final, we find different drugs based on novel drug targets and discuss the druggability (Fig 1). Finally, we search for different drugs based on new drug targets and discuss their drug availability through network pharmacology and molecular docking methods.

## 2. Method

### 2.1. SLE and proteins of CSF and plasma data source

In this study, CSF pQTL was selected from Yang’s study, which recruited 713 proteins from Washington University School of Medicine in St. Louis [13]. Plasma pQTL was obtained from Cheng’s study containing 1699 proteins from summary of 5 GWAS [5]. 4907 proteins and 18084 pQTL from the deCODE database which recruited 35,559 Icelanders from 24 August 2000 until 11 January 2019, were used for replication verification [14]. SLE was selected from James’ study whose participants were mostly from southern Europe (ncase = 5201; ncontrol = 9066) for MR analysis [15]. For replication verification, summary statistics were from Wang’s study whose participants came from the same continentas above (ncase = 4222, ncontrol = 8431) [16]. If the effect allele frequency was missing, the matched human genome build was used to fill in the missing.

### 2.2. Mendelian randomization analysis

Mendelian randomization analysis is an observational study way. For the selection criteria of IV: (1) SNP from cis-pQTL. (2) Significantly correlated with proteins (P<5×10^−8^). To avoid bias introduced by weak instruments of screening [17], we used F-statistic values to calculate the strength of IV. F > 10 were considered to be strongly correlated, satisfying the MR assumption a. (3) Independence (LD clumping r^2^ < 0.001), (4) Exclusion of pQTL within the major histocompatibility complex (MHC) region (chr6, from 26 Mb to 34 Mb).

According to the above selection criteria, we obtained 154 cis-pQTL of 154 CSF proteins and 738 cis-pQTL of 734 plasma proteins. We used "TwoSampleMR" (https://github.com/MRCIEU/TwoSampleMR) to perform two-sample MR analysis. Proteins were used as exposure, and SLE as outcome. If the proteins in MR analysis have only one SNP, the "Wald ratio" method will be used. Meanwhile, "Inverse variance weighted MR (MR-IVW)" was used for proteins with multiple SNPs. In addition, for screened SNPs that meet the above criteria, heterogeneity and pleiotropy tests were performed further. Results were adjusted using Bonferroni correction, and P<0.05/888 (5.63×10^−5^) were selected for subsequent analysis.

### 2.3. Reverse causality detection

Steiger filtering was employed to analyze the directionality between proteins and SLE, ensuring the credibility of the results and identifying reverse associations. Besides, we exchanged the exposure and outcome data to perform a secondary analysis in order to more accurately identify reverse causality: SLE was used as the exposure, and data from the original protein literature was obtained as the outcome.

What counts is that IVs were screened using the criteria described above. MR analysis was performed using MR-IVW, MR-Egger, weighted median, simple mode, and weighted mode. Statistical significance was achieved when P<0.05.

### 2.4. Colocalization analysis

Bayesian colocalization analysis can assess whether proteins and diseases are caused by the same pQTL, eliminating LD bias in MR analysis. We used the “coloc” package (https://github.com/chr1swallace/coloc) for colocalization analysis. Colocalization analysis contains five hypotheses: posterior probability (PP0), functional association only (PP1), GWAS association only (PP2), independent functional/GWAS associations (PP3), or colocalized functional/GWAS associations (PP4) [18]. By obtaining all pQTL data from the original article. We performed colocalization analysis of all SNPs within 1MB of the cis-pQTL obtained from the MR analysis. Finally, we use the result of the coloc.abf algorithm, PPH4 > 0.8, as a basis for co-localization in this study.

### 2.5. Phenotype scanning

We also further performed "phenome scans" to exclude confounding factors from the results. PhenoScanner V2 was used to search for associations of the IVs with other traits by searching previous GWAS [19]. As described above, IVs that did not satisfy MR assumptions b and c were excluded. Exclusion criteria: significant genome-wide association (p<5×10^−8^), IV associated with known risk factors.

### 2.6. Replication verification

To better verify the authenticity of the primary findings, data from different databases were used to repeat the experiments. Due to less CSF pQTL data, the above data were still used. Plasma proteins from the deCODE database were used as exposure. SLE from Wang’s study was used as the outcome [16]. The screen of IVs and MR analyses were consistent with the above. Because of just validation of preliminarily identified proteins, the range of P values was appropriately relaxed to P<0.05 to be considered significant.

### 2.7. Plasma-CSF proteins correlation

While both plasma and CSF proteins undergo metabolic circulation within the body, they possess distinct differences owing to the presence of the blood-brain barrier. To assess the discrepancy between proteins pQTL in plasma and CSF, we conducted Spearman correlation analysis to investigate the shared pQTL correlation between plasma and CSF. In addition, we set different p-value thresholds to explore whether the correlation changes with increasing significance levels.

### 2.8. Drug candidate confirmation

The proteins identified by the above screen series proved causally related to SLE. To explore the interactions between those SLE-associated proteins and the targets for medications already on the market, we searched drug databasea to find medications targeting the identified potential causal proteins including DrugBank (https://go.drugbank.com/), ChEMBL (https://www.ebi.ac.uk/chembl/), and Therapeutic Target Database (TTD) (https://idrblab.net/ttd/) [20].

### 2.9. Network pharmacology analysis

Active components of Voclosporin and Cyclosporine were searched by the Drugbank database. We obtained disease-related targets of SLE by employing the keyword "Systemic Lupus Erythematosus" in the Genecards (https://www.genecards.org/). Then the collected drug targets and SLE targets were calculated to receive overlapping targets in Venny 2.1.0 (https://bioinfogp.cnb.csic.es/tools/venny/). This study imported the identified shared targets into the STRING Database (https://string-db.org/) whose confidence was set to medium (0.400) to ascertain hub drug and disease targets, subsequently visualizing the results in the form of a PPI network. We performed the MCC algorithm to calculate the hub targes of drug candidates against SLE in the Cytohubba plugin of CytoScape, and constructed the related protein target network.

### 2.10. Molecular docking

Drug molecule structures of candidate drugs were downloaded from the ChemSpider database (https://www.chemspider.com/Default.aspx), and the target protein from the UniProt Database. Next, we dehydrated and then removed the active center ligands for the drug structure in PyMOL 2.6.0 software, and carried out hydrogenation and charge calculation for the target proteins in Autodock Tools 1.5.7 software. The molecular docking results of the key targets of SLE and the corresponding active components were visualized by Pymol.

## 3. Results

### 3.1. MR analysis

The causal relationship of CSF and plasma proteins on SLE were assessed using two-sample MR analysis. At Bonferroni correction (p<0.05/888), the result revealed that four potential causal proteins were finally obtained for SLE (Table 1 and S2 Fig). Intercellular adhesion molecule 1 (sICAM-1)(P = 4.62×10^−5^, OR = 0.49(0.35, 0.69)), Low affinity immunoglobulin gamma Fc region receptor II-b (FCG2B) (P = 7.63×10^−11^, OR = 0.57(0.48, 0.67)), and Protein phosphatase 3 catalytic subunit alpha (PPP3CA); Calcineurin subunit B type 1 (PPP3R1)(P = 5.47×10^−7^, OR = 0.66(0.57, 0.78)), Intercellular adhesion molecule 1 (ICAM1)(P = 4.62×10^−5^, OR = 0.90(0.86, 0.95)). Although sICAM-1 in CSF and ICAM-1 in plasma are same protein, they distribute in different microenvironments. Subsequent analyses will also be treated separately to distinguish whether this protein differs in CSF and plasma. By checking the original literature, PPP3CA; PPP3R1 corresponds to Calcineurin, so it is placed in one group. Increasing these four proteins will decrease the risk of SLE. No heterogeneity and pleiotropy were detected in the preliminary analyses.

**Table 1:** MR results for plasma and CSF proteins after Bonferroni correction.

Tissue	Protein	SNP	Outcome	Method	Beta	SE	P value	PVE	F	Steiger pval	OR(95% CI)
Plasma	PPP3CA; PPP3R1	rs17266357	SLE	Wald ratio	-0.41	0.08	5.47E-07	6.29%	66.9	6.15E-11	0.66(0.57, 0.78)
Plasma	ICAM1	rs5498	SLE	Wald ratio	-0.1	0.03	4.62E-05	70.18%	7769.9	0	0.90 (0.86, 0.95)
CSF	sICAM-1	rs5498	SLE	Wald ratio	-0.72	0.18	4.62E-05	57.39%	1124.84	1.04E-158	0.49 (0.35, 0.69)
CSF	FCG2B	rs4657041	SLE	Wald ratio	-0.57	0.09	7.63E-11	47.57%	757.73	9.53E-110	0.57 (0.48, 0.67)

• PVE = proportion of variance explained.

• F-value assesses the bias of instrumental variables; when F > 10, weak instrumental variables do not exist.

### 3.2. Reverse causality detection and Replication verification

**Fig 2:**
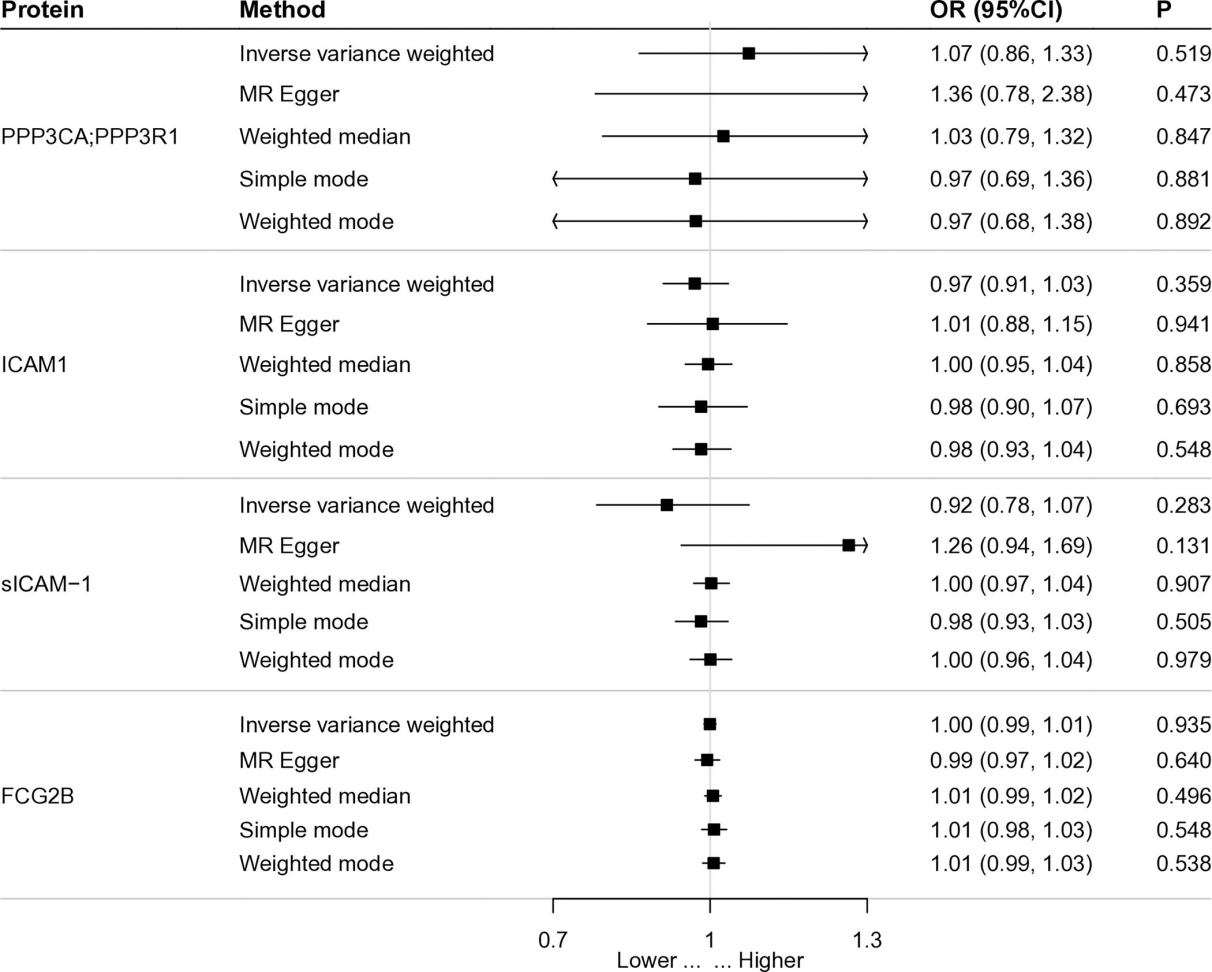
Forest map of reverse causality detection about significant protein.

Steiger filtering obtained results confirming the unidirectionality of causality. When exposure and outcome were interchanged, reverse MR analysis showed that SLE was no causality for the four proteins, which further ensured directionality (Fig 2). Using data from different databases and the same analytical method, the result display that PPP3CA;PPP3R1 (P = 3.11×10^−7^, OR = 0.61), and FCG2B (P = 3.91×10^−5^, OR = 0.68) were re-validated a causal effect for SLE (S1 Table). Meanwhile, increasing these two proteins reduced the risk of SLE, consistent with the results of previous analyses.

### 3.3. Colocalization analysis and phenotype scanning

We subsequently performed the co-localization analysis of the four proteins obtained from MR analysis. The proteins were tested by the coloc.abf algorithm to identify if they shared a common causal variant with SLE. The results strongly suggest that PPP3CA;PPP3R1 (PH4.coloc.abf = 0.872), FCG2B (PH4.coloc.abf = 0.985), and SLE share the same variants (S2 Table).

Finally, confounders were removed by phenotypic scanning (S3 Table). FCG2B (rs4657041) was found to be associated with systemic lupus erythematosus, inflammatory bowel disease, ulcerative colitis, and low affinity immunoglobulin gamma fc region receptor II-a/b. Because FCG2B (rs4657041) was directly associated with SLE, it did not meet MR assumption a. So it was not included in the analysis of drug-targeted proteins. sICAM-1 (rs5498) and ICAM-1 (rs5498) were associated with White blood cell count, soluble intercellular adhesion molecule 1 ICAM 1, Intercellular adhesion molecule 1, etc. PPP3CA;PPP3R1 (rs17266357) correlated with mean platelet volume, calcineurin.

### 3.4. Correlation and drug targets analysis

There was a non-significant negative correlation between the CSF and plasma proteins MR results (Spearman correlation coefficient = −0.035). When restricting the number of proteins included in the analysis with different P-value thresholds, a negative correlation was still present and remained insignificant (S3 Fig). Through a series of analyses, it was finally shown that PPP3CA;PPP3R1 are genetically linked to SLE. Look for the drug records of the proteins in the database. Three PPP3R1 target proteins were looked up in the DrugBank, including Calcineurin subunit B type 1, Calcineurin subunit B type 2, and Calcium and integrin-binding protein 1. For PPP3R1, five drugs have been approved, and two drugs have explicit pharmacological action (Table 2). Interestingly, Voclosporin is a potential drug target for both causal proteins, which has clear pharmacological action and has been approved on the market.

**Table 2:** Drug targets analysis.

Protein	Targe	Drug name	Source	Drug group	Pharmacological action	Actions
PPP3R1	Calcineurin subunit B type 1	Voclosporin	DrugBank	approved, investigational	yes	inhibitor
	Calcineurin subunit B type 2	Cyclosporine	DrugBank	approved,investigational	yes	inhibitor
		Voclosporin	DrugBank	approved, investigational	yes	inhibitor
PPP3CA	-	Voclosporin	ChEMBL; TTD	approved, investigational	yes	inhibitor

### 3.5. Network pharmacology analysis

**Fig 3:**
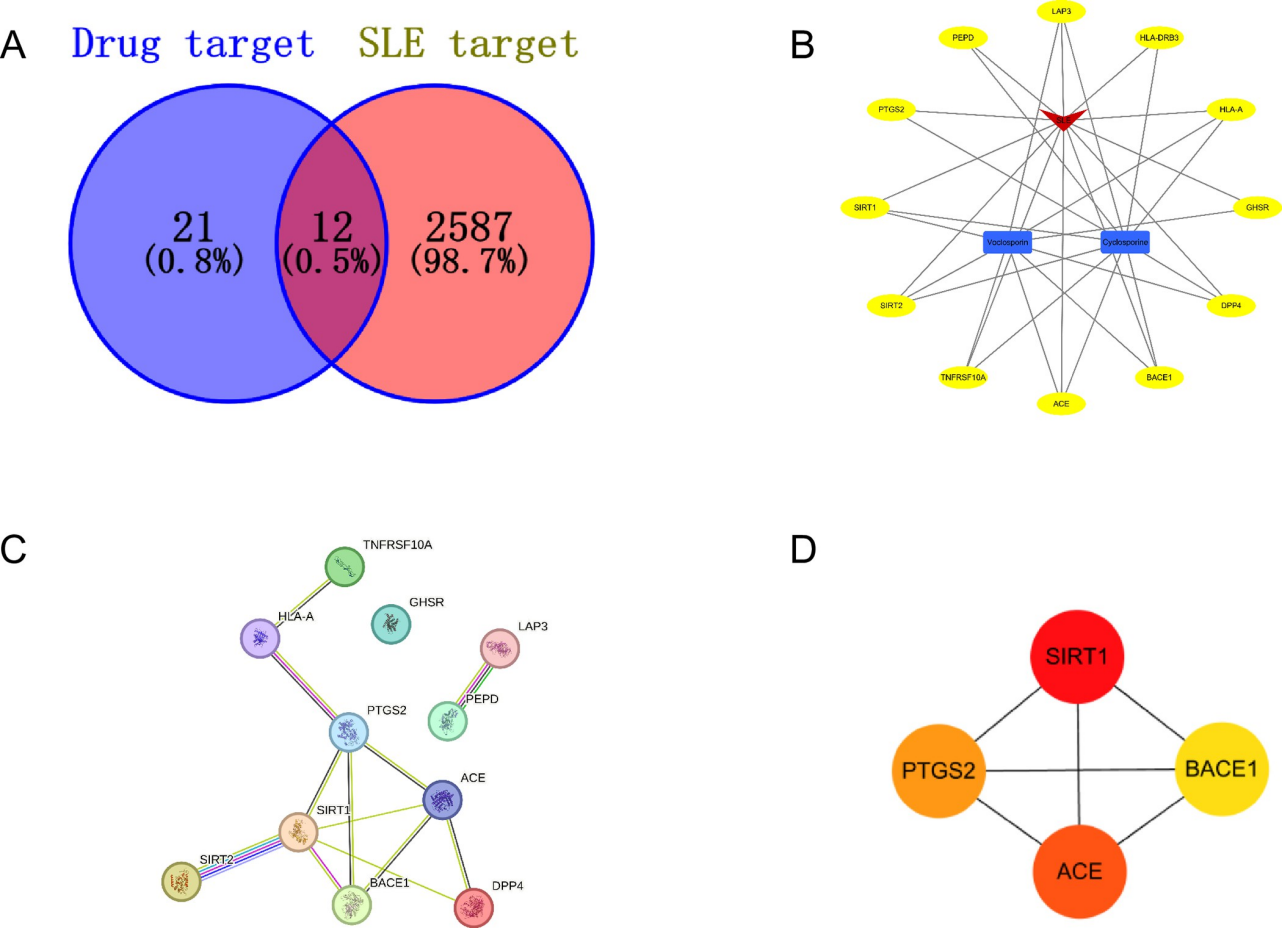
Network pharmacology analysis. (A) Drug-disease target Venn diagram; (B) Drug-Disease-Target network. The red color indicates SLE; The blue color indicates the drugs; the yellow color indicates the targets; (C) PPI network of shared targets of Voclosporin and Cyclosporine in treatment of SLE; (D) Core targets of overlapping targets.

33 drug targets were screened in Drugbank and 2599 targets of SLE in the Genecards Database. The Venn plots show that SLE targets and Voclosporin and Cyclosporine targets have shared 12 common ones (Fig 3A). Next, we draw targets of drug candidates into a drug disease-target network diagram to show intuitively this relationship (Fig 3B). The PPI network by feeding 12 overlapping targets into the STRING database revealed 11 nodes,12 edges, and 2.18 average node degree (Fig 3C). Then we imported the PPI network data from the STRING platform into Cytoscape to calculate and select the top 4 core targets with the Cytohubba plug-in (Fig 3D). The results of algorithm reveal that SIRT1, ACE, PTGS2 and BACE1 may play important roles in the treatment of SLE.

### 3.6. Molecular docking analysis

**Fig 4:**
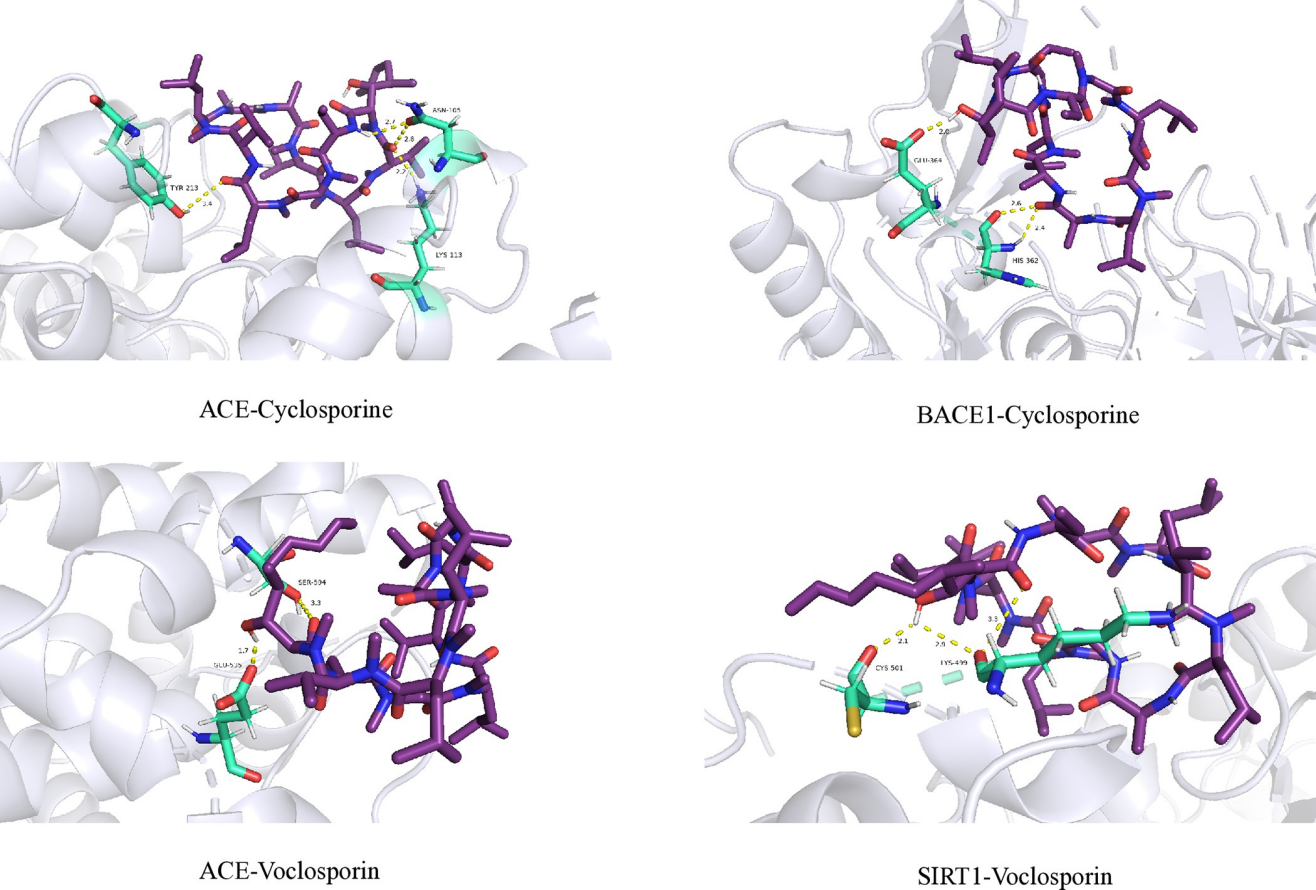
Molecular docking diagrams of SLE related targets with main compounds of Voclosporin and Cyclosporine.

The key targets SIRT1 (PDB: 4I5I), ACE (PDB: 1O86), and BACE1 (PDB: 1TQF) with high degree values in the PPI network were selected for molecular docking verification with Voclosporin and Cyclosporine. The results of Molecular docking analysis exhibited that bioactive compounds of Voclosporin and Cyclosporine could be bound into the docking pocket, and demonstrated strong docking activity (Table 3; Fig 4). Cyclosporine bound to ACE by forming hydrogen bonds with TYR-213 (length: 3.4 Å), ASN-105 (2.8 Å), ASN-105 (2.7 Å) and LYS-113 (2.2 Å). Voclosporin bound to SIRT1 by forming hydrogen bonds with LYS-499 (2.9 Å), CYS-501 (2.1 Å) and LYS-499 (3.3 Å).

**Table 3:** Docking simulation for active molecular and targets of SLE.

Molecular name	Targets	PBD ID	Residue involved in H bonding	Docking score (kcal/mol)
Cyclosporine	ACE	1O86	TYR-213; ASN-105; LYS-113	-2.59
Voclosporin	SIRT1	4I5I	LYS-499; CYS-501	-1.97
Cyclosporine	BACE1	1TQF	GLU-364; HIS-362; HIS-362	-0.19
Voclosporin	ACE	1O86	SER-594; GLU-535 (1.7 Å)	-0.17

## 4. Discussion

In this study, we used CSF and plasma proteins for MR analysis and co-localization analysis to find proteins causally associated with SLE. We used cis-pQTL as IV because it directly affects transcription and translation, and reduces horizontal pleiotropy [21]. Preliminary analyses of the proteins provided four causal proteins. At the same time, we found one identical protein, ICAM1, in CSF and plasma. Because the results of MR analysis may be affected by reverse causality, we corroborated each other by Steiger filtering and reverse causality detection to minimize the error. The results showed that the proteins analyzed by MR did not have reverse causality. Bayesian co-localization analysis was performed with a threshold of 0.8 to exclude the bias introduced by LD. In the co-localization analysis, although ICAM1 was a failure, it could not be ignored in explaining the mechanism of the disease. When searching for relevant phenotypes for instrumental variables, FCG2B was considered a confounder in the MR analysis because it was directly associated with SLE. Finally, we found that PPP3CA and PPP3R1 are causal proteins of the disease that can be potential drug targets. For PPP3CA and PPP3R1, the target drug is Voclosporin. It has been clearly available for the treatment of lupus nephritis. Then, the therapeutic effects of Voclosporin and Cyclosporine on SLE were evaluated by network pharmacology and molecular docking methods.

ICAM-1, a cell surface glycoprotein and an adhesion receptor, is involved in antigen presentation, immune cell initiation, leukocyte transport, and effector functions [22]. In addition, it is involved in regulating endothelial and epithelial barriers and influences tumor development and metastasis [23]. ICAM-1 has been implicated in a variety of diseases. FCG2B is engaged in a variety of effects and regulation, including phagocytosis of immune complexes and regulation of B cell antibodies. Kyogoku suggests that FCG2B is implicated in SLE [24].

By searching the original literature [25], PPP3CA; PPP3R1 targets Calcineurin (CaN). CaN, primarily located in the cytosol, is a serine/threonine phosphatase needing calcium and calmodulin (CaM) [26]. CaN and CaM bind calcium, and then the two bind to each other to form an active phosphatase. This way couples calcium signaling to dephosphorylation of proteins [27]. CaN activates the nuclear factor family of T cells, regulates a number of receptors and channels, and regulates mitochondria-associated proteins and microtubule-associated proteins [26]. The best known of these is that CaN activates T cells and drives the immune system. Therefore, calcineurin inhibitors (FK506 (tacrolimus), and cyclosporin A) are used as immunosuppressants including organ transplantation [28]. Calcineurin inhibitors have been used to treat autoimmune diseases with some adverse effects, possibly due to some effects in non-immune tissues [28]. Therefore, it is crucial to clarify the molecular biological mechanism of CaN as well as the substrates for the reaction. PPP3CA is calmodulin-stimulated protein phosphatase [29]. Several studies have shown that variants in PPP3CA lead to epilepsy [30, 31]. Maria showed that PPP3CA is associated with SLE disease subtypes [32]. PPP3R1 is a regulatory subunit of calcineurin. It is thought to be associated with Alzheimer’s disease [33, 34].

Angiotensin-converting enzyme (ACE) plays a key role in the regulation of blood pressure by converting angiotensin I to the potent vasoconstrictor angiotensin II [35]. At present, the relationship between ACE and SLE has been studied, especially the polymorphism of the ACE gene. The DD genotype (associated with higher ACE activity levels) was found to be more frequent in patients with SLE and associated with visceral damage in this population [36]. This suggests that the ACE gene I/D polymorphism may affect the pathological process of SLE and may be involved through angiotensin II-mediated inflammatory and immunomodulatory mechanisms. Silent information regulator 2 related enzyme 1 (SIRT1) is a NAD+ -dependent histone deacetylase with regulatory effects on a variety of physiological processes including immune responses, which may be related to the development of SLE [37]. Related studies have shown that the long non-coding RNA MALAT-1 plays a role in the pathogenesis of SLE by regulating SIRT1 expression, suggesting a possible relationship between SIRT1 and the development of SLE [38]. BACE1, β-secretase 1, plays A key role in the production of β-amyloid peptide (Aβ) by cleaving amyloid precursor protein (APP). It is best known for its function in promoting the accumulation of Aβ peptides in the pathogenesis of Alzheimer’s disease [39]. However, the activity of BACE1 is not limited to the brain, it is identified in various cell types and implicated in a variety of physiological and pathological processes, including metabolic diseases and obesity [40]. Although the direct relationship between BACE1 and SLE has not been extensively studied, the broad effects of BACE1 on immune function and cellular signaling pathways imply a potential intersection with autoimmune diseases such as SLE.

Although this study did not find SLE-associated proteins from those analyzed CSF proteins, it was because the available pQTL data in the GWAS data of CSF proteins were limited. For example, PPP3CA;PPP3R1 did not exist in this CSF GWAS data, but the utility of PPP3CA;PPP3R1 in SLE in CSF cannot be ruled out. Neuropsychiatric SLE (NPSLE) means that the lesions involve the nervous system, which may affect 12% ~ 95% of patients with SLE. Due to the existence of the blood-brain barrier, research on PPP3CA;PPP3R1 protein for NPSLE has not been carried out and is relatively scarce. For future research, we will focus on the dual role of PPP3CA;PPP3R1 in CSF and plasma and explore the impact of the blood-brain barrier on target drug. We hope future research can conduct more basic and animal experiments.

The strength of our study lies in the use of MR analysis and co-localization analysis, which eliminates the effects of confounding factors and reverses causation as much as possible. Using cis-pQTL as an instrumental variable ensured robustness between SNP and proteins. Demonstrate the reproducibility of the results by replication validation. Avoid the chance of results due to differences between data. In addition, using large samples of GWAS data, there is enough statistical strength to prove the relationship between proteins and diseases. AND network pharmacology and molecular docking technology were used to evaluate the efficacy of target drugs in the treatment of SLE. Nonetheless, the study has some limitations. While cis-pQTL is an ideal tool for analyzing drug targets, this would also ignore some of the roles of trans-pQTL in disease. Since the population is limited to Europe, the results may be biased relative to other regions. In addition, information related to network pharmacology is obtained through online databases, and the database information still needs to be further improved.

## 5. Conclusions

By using MR analysis, we identified proteins that have a causal relationship with SLE. These proteins are essential in explaining the disease’s mechanisms and molecular pathways. Finally, we discuss the role of PPP3CA, and PPP3R1 in SLE and potential drug use. Based on data mining network pharmacology, and molecular docking method validation, it provides new evidence for potential drug as an SLE treatment, and warrants further clinical investigation.

## Supporting information

S1 Fig Study flowchart of the Mendelian randomization study. (TIF)

S2 Fig Volcano plot of MR results for plasma and CSF proteins. (TIF)

S3 Fig Correlation of MR results for plasma and CSF proteins. (TIF)

S1 Table Result of replication verification. (CSV)

S2 Table Result of colocalization analysis. (CSV)

S3 Table Result of phenotype scanning. (CSV)

## Abbreviations

SLE: Systemic lupus erythematosus
MR: Mendelian randomization
pQTL: protein quantitative trait loci
GWAS: published genome-wide association studies
CSF: cerebrospinal fluid
PPI: Protein-protein interaction
LD: linkage disequilibrium
NSAIDs: nonsteroidal anti-inflammatory drugs
sICAM-1: Intercellular adhesion molecule 1
FCG2B: Fc region receptor II-b
PPP3CA: Protein phosphatase 3 catalytic subunit alpha
PPP3R1: Calcineurin subunit B type 1
ACE: Angiotensin-converting enzyme
SIRT1: Silent information regulator 2 related enzyme 1
APP: amyloid precursor protein
