# Supplementary figures and images for: Exploring potential drug targets for SLE through Mendelian randomization and network pharmacology
Source: PLoS One. 2025 Jan 17;20(1):e0316481. doi: 10.1371/journal.pone.0316481 (PMC11741580; doi:10.1371/journal.pone.0316481)

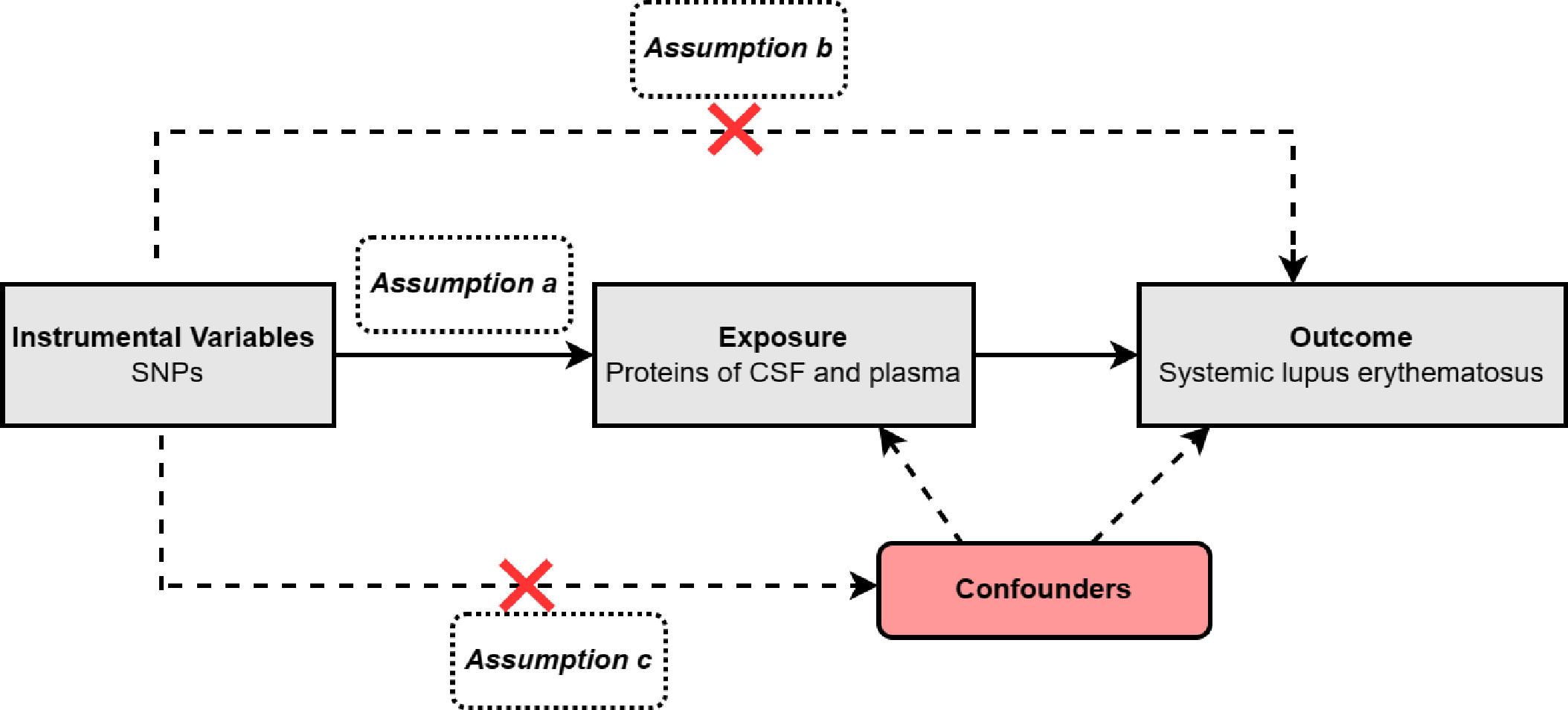

Supplement: S1 Fig — (TIF) [file pone.0316481.s001.tif]

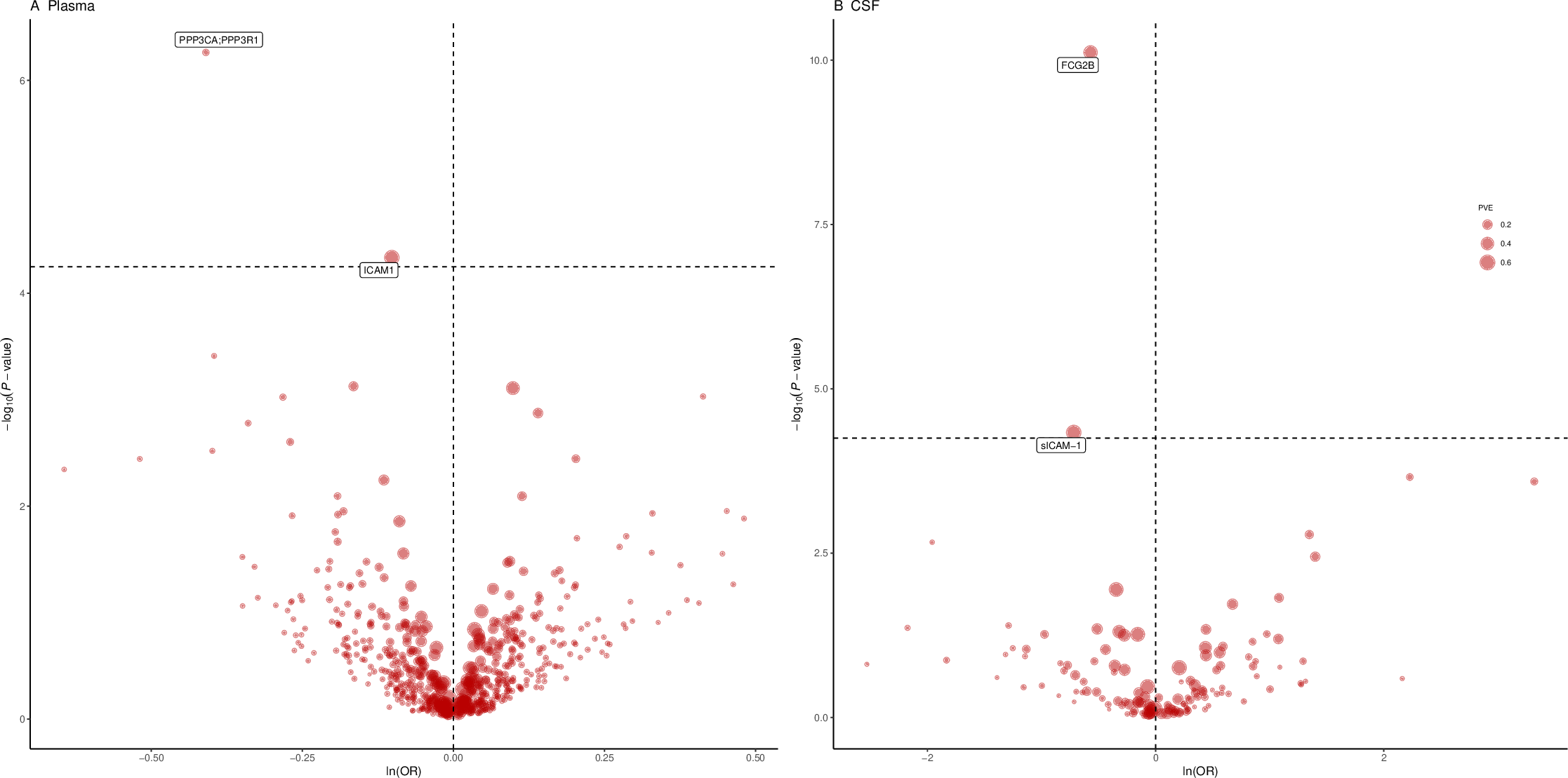

Supplement: S2 Fig — (TIF) [file pone.0316481.s002.tif]

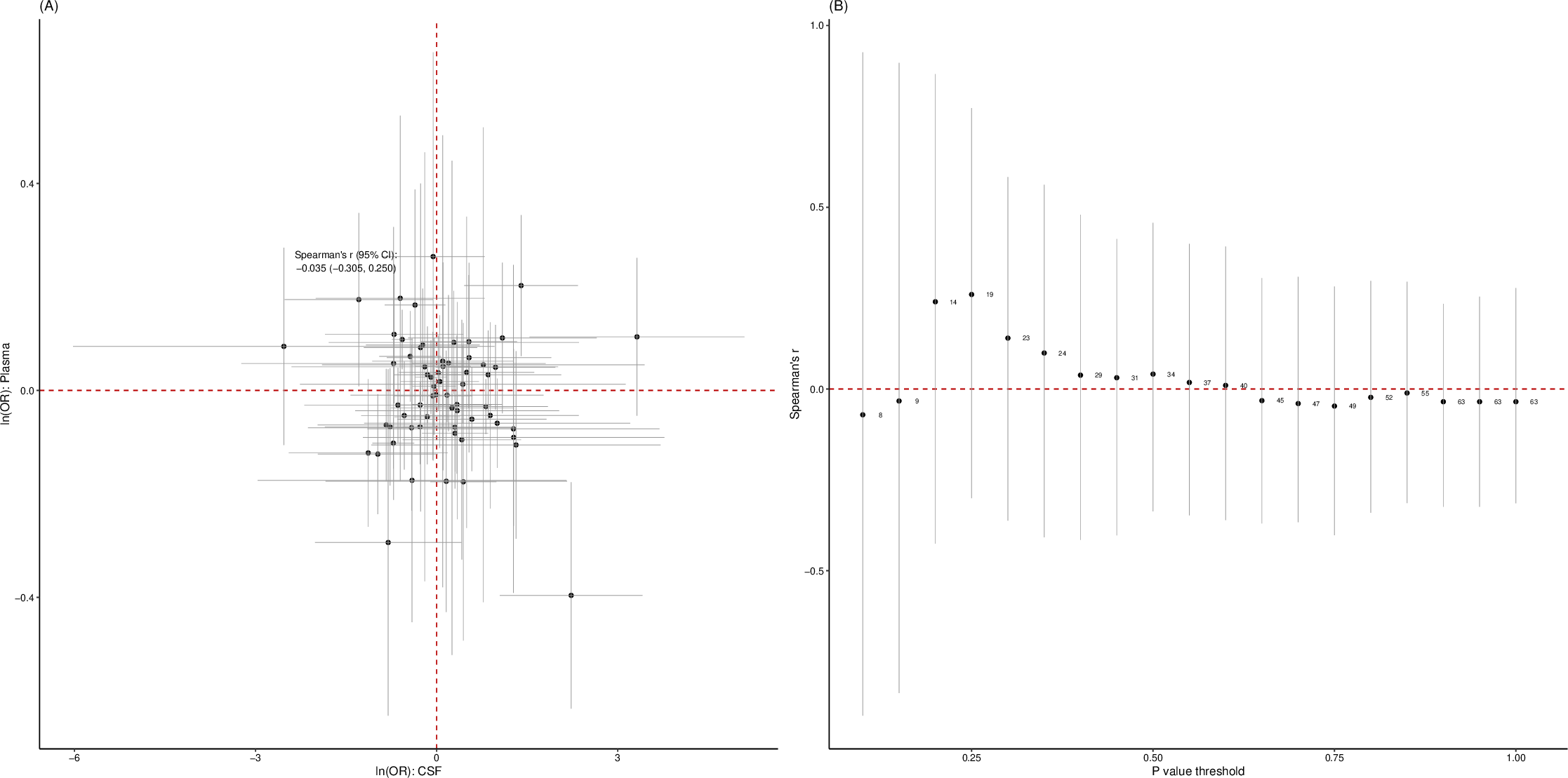

Supplement: S3 Fig — (TIF) [file pone.0316481.s003.tif]
